# *Pleurotus ostreatus* opposes mitochondrial dysfunction and oxidative stress in acetaminophen-induced hepato-renal injury

**DOI:** 10.1186/1472-6882-14-494

**Published:** 2014-12-15

**Authors:** Yahya M Naguib, Rania M Azmy, Rehab M Samaka, Mohamed F Salem

**Affiliations:** Department of Clinical Physiology, Faculty of Medicine, Menoufia University, Menoufia, Egypt; Department of Medical Biochemistry, Faculty of Medicine, Menoufia University, Menoufia, Egypt; Department of Pathology, Faculty of Medicine, Menoufia University, Menoufia, Egypt; Genetic Engineering and Biotechnology Research Institute, Sadat City University, Menoufia, Egypt

**Keywords:** Pleurotus ostreatus, Oxidative stress, Acute hepato-renal injury, Mitochondrial dysfunction, Acetaminophen, Antioxidant

## Abstract

**Background:**

Acetaminophen (APAP)-induced toxicity is a predominant cause of acute hepatic and renal failure. In both humans and rodents toxicity begins with a reactive metabolite that binds to proteins. This leads to mitochondrial dysfunction and nuclear DNA fragmentation resulting in necrotic cell death. Pleurotus ostreatus (an edible oyster mushroom) is well recognized as a flavourful food, as well as a medicinal supplement. In the present study, we evaluated the role of Pleurotus ostreatus in the protection against APAP-induced hepato-renal toxicity. We also explored the mechanism by which Pleurotus ostreatus exerts its effects.

**Methods:**

Ninety adult male Swiss albino mice were divided into three groups (30 mice/group). Mice were offered normal diet (control and APAP groups), or diet supplemented with 10% Pleurotus ostreatus (APAP + Pleurotus ostreatus) for 10 days. Mice were either treated with vehicle (control group, single intra-peritoneal injection.), or APAP (APAP and APAP + Pleurotus ostreatus groups, single intra-peritoneal injection, 500 mg/kg), 24 hours after the last meal.

**Results:**

APAP increased serum levels of alanine aminotransferase (ALT), aspartate aminotransferase (AST) glutamate dehydrogenase (GDH), creatinine, blood urea nitrogen (BUN), urinary kidney injury molecule-1 (KIM-1), and hepatic and renal malondialdehyde (MDA) content. APAP decreased hepatic and renal glutathione (GSH) content, as well as glutathione peroxidase (GSH-Px) and superoxide dismutase (SOD) activities. Supplementation with Pleurotus ostreatus significantly reduced APAP-induced elevated levels of ALT, AST, GDH, creatinine, BUN, KIM-1and MDA, while GSH level, and GSH-Px and SOD activities were significantly increased. Our findings were further validated by histopathology; treatment with Pleurotus ostreatus significantly decreased APAP-induced cell necrosis in liver and kidney tissues.

**Conclusions:**

We report here that the antioxidant effect of Pleurotus ostreatus opposes mitochondrial dysfunction and oxidative stress accompanying APAP over-dose, with subsequent clinically beneficial effects on liver and kidney tissues.

## Background

Acetaminophen (N- acetyl-p-aminophenol, APAP) is a widely prescribed non-narcotic analgesic and antipyretic drug. APAP is commonly sold in the clinic as well as numerous over-the-counter preparations either as a single compound or in combination with other drugs [1–3]. APAP is metabolized by cytochrome P450 (CYP) to form the highly reactive species, *N*-acetyl-*p*-benzoquinone imine (NAPQI), which under normal conditions is readily detoxified by conjugation with glutathione (GSH). However, an overdose of APAP can lead to severe liver and/or kidney injury in humans and in experimental animals [4, 5]. Most importantly, in the presence of hepatic, renal or cardiopulmonary insufficiency, even therapeutic doses of APAP may cause hepato-renal damage [6, 7]. High doses of APAP saturate the detoxification pathways [8]; depletion of GSH leaves NAPQI free to bind to possibly critical cellular proteins and cause cell necrosis. Therefore, APAP toxicity is determined by the amount of NAPQI produced and the insufficient availability of GSH for APAP detoxification [9–11].

Reactive oxygen species (ROS) are fundamentally correlated to oxidative stress. ROS have been implicated in a number of disease processes, including hepatic injury, renal injury, cardiac diseases, neurodegenerative diseases, diabetes, pulmonary diseases as well as cancer [12–20]. Maintaining the balance between the production of ROS and the availability of antioxidant enzymes, such as superoxide dismutase (SOD), catalase (CAT), and glutathione peroxidase (GPx), is consequently critical. This could be an important mechanism for preventing the oxidative stress-induced tissue damage. ROS-antioxidants balance has been suggested to have an important role in the development of APAP toxicity [5]. Lipid peroxidation, mediated by ROS, is believed to be an important cause of cell membranes damage. The role of ROS in mediating the microvascular disturbances that precede tissue damage induced by various chemicals has gained much attention. It has been shown that, during APAP intoxication in the mouse, toxic ROS are generated and actively participate in the pathophysiological process leading to hepatocyte necrosis [4].

Scientific and clinical interests have risen towards the use of mushrooms with potential therapeutic effects. Edible mushrooms are a valuable source of biologically active compounds. The medicinal potential of edible mushrooms arises from the fact that they are natural, less expensive and have minimal side effects. Mushrooms demonstrate their efficiency against numerous diseases and metabolic disturbances. These therapeutic effects seem to be underlined by multiple complex cellular and molecular actions [21]. Pleurotus ostreatus (an oyster mushroom) is one of the widely cultivated edible mushrooms. Pleurotus ostreatus demonstrated antioxidative, hypocholesterolemic, and antiatherogenic activities [22]. Antitumor properties [23], as well as the ability to enhance the immune system have also been reported [24].

In the present study, we aimed to evaluate the protective effects of Pleurotus ostreatus on APAP-induced hepato-renal toxicity in mice, with emphasis on mitochondrial dysfunction trying to elucidate the mechanism(s) by which Pleurotus ostreatus may execute its protective effect.

## Methods

### Animals

Ninety male Swiss albino mice, 10–14 weeks old weighing approximately 20–25 g, were used in the present study. Mice were maintained under controlled temperature, humidity, and 12 hour light/dark cycles. The animals were fed standard rodent chow and allowed free access to water *ad libitum*, and were kept for 10 days prior to any procedure to allow proper acclimatization. Animal care and use was approved by the Ethics Committee of Faculty of Medicine-Menoufia University-Egypt. The experiments were carried in accordance with the Guide for the Care and Use of Laboratory Animals published by the US National Institutes of Health (NIH Publication no. 85–23, revised in 1996).

After acclimatization, mice were divided randomly into the following groups (30 mice/group): (1) control group, (2) APAP group, and (3) APAP + Pleurotus ostreatus group. Mice in the control and APAP groups were fed normal rodent diet; while those in the APAP + Pleurotus ostreatus group were fed normal rodent diet supplemented with 10% Pleurotus ostreatus for 10 consecutive days. Mice were fasted for 12 hours before treatment with APAP or vehicle as indicated. Acute liver injury was induced in APAP and APAP + Pleurotus ostreatus groups by a single intra-peritoneal (i.p) injection of 500 mg/kg APAP (Sigma-Aldrich Co., Mo, USA) dissolved in warm phosphate-buffered saline (PBS, pH 7.4). Control mice were injected with equal volumes of the vehicle.

Five mice from each group were scarified either just before (0), or after (1, 2 and 8) hours following APAP or vehicle treatment. The aim of the separate experiment was to validate the anti-oxidant properties of Pleurotus ostreatus.

### Collection of oyster mushroom

Mature fruiting bodies of Pleurotus ostreatus were kind gift from Dr Mohamed F Salem (Genetic Engineering and Biotechnology Research Institute, Sadat City University, Egypt). The fruiting bodies were dried in sunlight and crushed into powder. The powder was mixed with the basal diet.

### Characterization of oyster mushroom dried powder

Protein, fat and ash contents were determined using standard analytical methods [25]. Total dietary fiber (TDF) constituted the sum of soluble and insoluble dietary fiber and was determined using enzymatic method [26]. Analytical determinations were conducted in three independent replications and the results are presented in grams per 100 g dry powder.

The amino acid composition was identified as described previously [27]. High performance liquid chromatography (HPLC) analysis was carried out in an Agilent 1220 Infinity system (Santa Clara, CA, USA). The amino acid composition was expressed as percentage of protein.

### Blood and tissue samples collection

All mice were scarified 24 hours after APAP injection. Blood was drawn from each mouse via cardiac puncture. The blood was allowed to coagulate for 30 minutes at room temperature. Blood samples were then centrifuged at 2000 rpm for 10 min to separate serum samples. Serum samples were stored at -20°C. Serum samples were used for the estimation alanine aminotransferase (ALT), aspartate aminotransferase (AST), glutamate dehydrogenase (GDH), creatinine and blood urea nitrogen (BUN).

The liver and kidneys were carefully dissected from the fat and connective tissue. The tissues were rinsed several times with cold saline and air dried on filter paper. Liver and kidney specimens were used for the preparation of tissue homogenates and tissue slides for Haematoxylin and Eosin (Hx & E) stain.

### Urine samples collection

Urine samples from the mice were collected on day 1 after disease induction. 24-hour urine collection using metabolic cages was carried out for all mice. All collected urine samples were aliquoted and frozen away.

### Preparation of tissue homogenates

Specimens from the liver and kidney were weighted and homogenized separately with tissue homogenizer (MPW120, MPW Medical Instruments, China). For estimation of tissue glutathione (GSH), malondialdehyde (MDA) levels and the activities of glutathione peroxidise (GSH-Px), tissues were homogenized in phosphate buffer saline (PBS) 50 mM pH 7.4. For estimation of superoxide dismutase (SOD) tissues were homogenized in potassium phosphate buffer (PPB) 10 mM pH 7.4. The crude tissue homogenate was centrifuged at 10,000 rpm, for 15 minutes in ice-cold centrifuge, and the resultant supernatant was collected and stored at -20°C.

### Measurement of APAP protein adducts in liver tissue homogenate

Measurement of APAP-cysteine (APAP-CYS) in liver tissue homogenate was performed using high pressure liquid chromatography with electrochemical detection (HPLC-ECD) as described previously [28].

### Biochemical analysis

Serum levels of alanine aminotransferase (ALT), aspartate aminotransferase (AST), creatinine and blood urea nitrogen (BUN) (ELITech, France), and glutamate dehydrogenase (GDH) (QuantiChrom™, BioAssay Systems, USA) were determined by routine kinetic and fixed rate colorimetric methods on a Jenway Genova autoanalyser (UK) [29–31].

Urinary kidney injury molecule-1 (KIM-1) was measured by a highly sensitive two-site enzyme linked immunoassay (ELISA) (ALPCO Diagnostics, USA) [32, 33].

Tissue levels of glutathione (GSH) and malondialdehyde (MDA) (QuantiChrom™, BioAssay Systems, USA), glutathione peroxidase (GSH-Px) and superoxide dismutase (SOD) (EnzyChrom™, BioAssay Systems, USA), were determined by colorimetric method [34–37].

### Haematoxylin and Eosin (Hx & E) stain

Specimens from the liver and kidney were fixed in 10% formol saline for 5–7 days. The specimens were washed in tap water for 10 minutes and then dehydrated in graded ethanol solutions (70%, 90% over night and 100% ethanol solution for three changes one hour each). The specimens were cleared in xylene (20–30 times). After that, specimens were impregnated in soft paraffin wax at 55–60°C for two hours then in hard paraffin wax at room temperature in moulds. Tissue blocks were cut into section of 5 microns thickness by using rotator microtome. Tissue sections were dipped in a warm water-bath, picked up on clean slides, and placed on hot plate for two minutes. Finally, tissue sections were stained with haematoxylin and eosin stain for general architecture of the studied tissues.

### Statistical analysis

Results are expressed as mean ± standard error (SE). Student t-test or repeated-measures Analysis of Variances (ANOVA) were used for statistical analysis of the different groups whichever appropriate, using Origin^®^ software and the probability of chance (p values). P values < 0.05 were considered significant.

## Results

**Figure 1:**
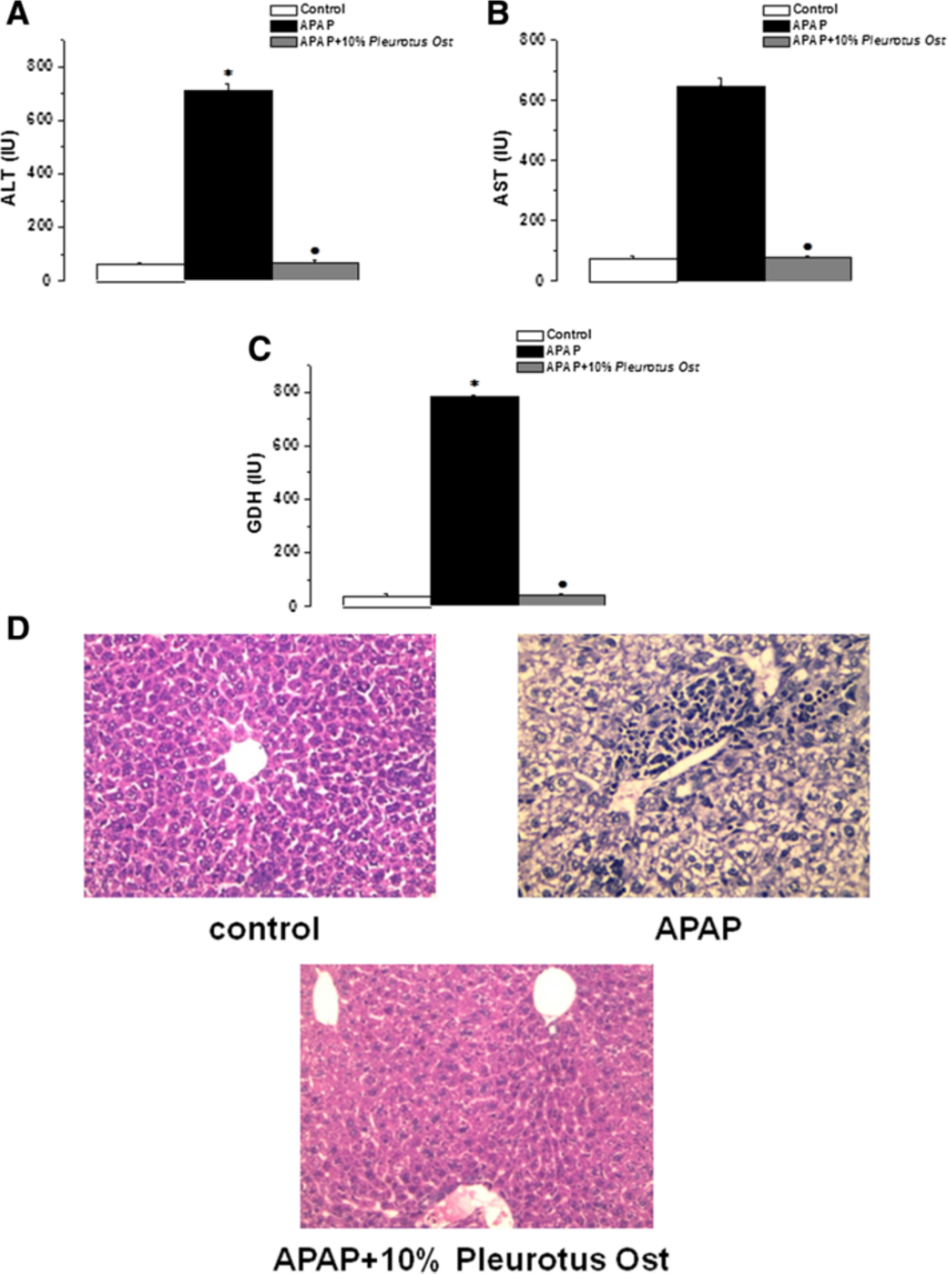
**Pleurotus ostreatus protects against liver injury in APAP-overdose treated mice. (A)** Serum ALT levels in control (white column), APAP treated (black column) and APAP + 10% Pleurotus ostreatus treated (grey column) groups. **(B)** Serum AST levels in control (white column), APAP treated (black column) and APAP + 10% Pleurotus ostreatus treated (grey column) groups. **(C)** Serum GDH levels in control (white column), APAP treated (black column) and APAP + 10% Pleurotus ostreatus treated (grey column) groups. **(D)** Representative photomicrograph of Hx & E stained liver sections from control, APAP treated and APAP + 10% Pleurotus ostreatus treated mice (×400). (Significant = p < 0.05, *significant when compared to the control group, • significant when compared to the APAP treated group. Number of mice = 10/group).

Serum ALT increased significantly in the APAP-treated group when compared to the control group (710.5 ± 25.8 vs 60.7 ± 8.2 IU, P < 0.05). Serum ALT levels were significantly lower in the APAP + Pleurotus ostreatus group than that in the APAP group (67.4 ± 8.2 IU, P < 0.05). There was no statistically significant difference in serum ALT between APAP + Pleurotus ostreatus and control groups (P > 0.05) (Figure 1A).

Serum AST increased significantly in the APAP-treated group when compared to the control group (645.7 ± 30.8 vs 72.4 ± 11.2 IU, P < 0.05). Serum AST levels were significantly lower in the APAP + Pleurotus ostreatus group than that in the APAP group (77.4 ± 8.3 IU, P < 0.05). There was no statistically significant difference in serum AST between APAP + Pleurotus ostreatus and control groups (P > 0.05) (Figure 1B).

Serum GDH increased significantly in the APAP-treated group when compared to the control group (782.6 ± 36.5 vs 38.9 ± 6.3 IU, P < 0.05). Serum GDH levels were significantly lower in the APAP + Pleurotus ostreatus group than that in the APAP group (41.7 ± 7.2 IU, P < 0.05). There was no statistically significant difference in serum GDH between APAP + Pleurotus ostreatus and control groups (P > 0.05) (Figure 1C).

The appearance of liver tissues 24 hours after APAP injection was confirmed by histopathological observation. Centrilobular necrosis, sinusoidal congestion, lymphocytes infiltration and Kupffer cells around the central vein, loss of cell boundaries and ballooning degeneration were observed after administration of acetaminophen. However, mice treated with 10% Pleurotus ostreatus preserved normal hepatic architecture with minimal changes (Figure 1D).

**Figure 2:**
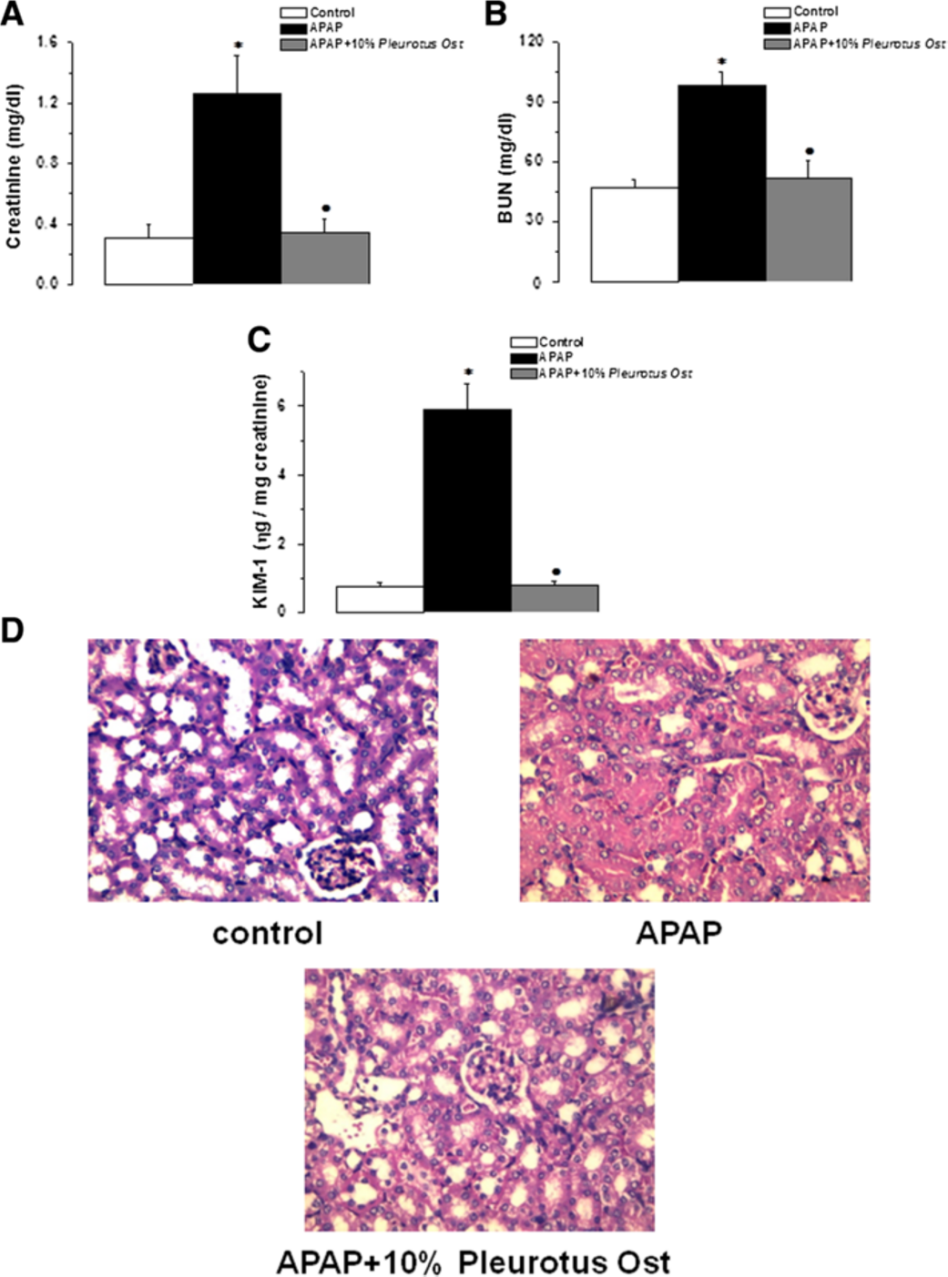
**Pleurotus ostreatus protects against kidney injury in APAP-overdose treated mice. (A)** Serum creatinine levels in control (white column), APAP treated (black column) and APAP + 10% Pleurotus ostreatus treated (grey column) groups. **(B)** Serum BUN levels in control (white column), APAP treated (black column) and APAP + 10% Pleurotus ostreatus treated (grey column) groups. **(C)** Urinary KIM-1 levels in control (white column), APAP treated (black column) and APAP + 10% Pleurotus ostreatus treated (grey column) groups. **(D)** Representative photomicrograph of Hx & E stained kidney sections from control, APAP treated and APAP + 10% Pleurotus ostreatus treated mice (×400). (Significant = p < 0.05, *significant when compared to the control group, • significant when compared to the APAP treated group. Number of mice = 10/group).

Serum creatinine increased significantly in the APAP-treated group when compared to the control group (1.26 ± 0.25 vs 0.31 ± 0.08 IU, P < 0.05). Serum creatinine levels were significantly lower in the APAP + Pleurotus ostreatus group than that in the APAP group (0.34 ± 0.09 IU, P < 0.05). There was no statistically significant difference in serum creatinine between APAP + Pleurotus ostreatus and control groups (P > 0.05) (Figure 2A).

Serum BUN increased significantly in the APAP-treated group when compared to the control group (98.4 ± 6.7 vs 47.3 ± 4.2 IU, P < 0.05). Serum BUN levels were significantly lower in the APAP + Pleurotus ostreatus group than that in the APAP group (51.7 ± 9.2 IU, P < 0.05). There was no statistically significant difference in serum BUN between APAP + Pleurotus ostreatus and control groups (P > 0.05) (Figure 2B).

Urinary KIM-1 increased significantly in the APAP-treated group when compared to the control group (5.91 ± 0.76 vs 0.78 ± 0.11 ng/mg creatinine, P < 0.05). Urinary KIM-1 levels were significantly lower in the APAP + Pleurotus ostreatus group than that in the APAP group (0.81 ± 0.18 ηg/mg creatinine, P < 0.05). There was no statistically significant difference in Urinary KIM-1 between APAP + Pleurotus ostreatus and control groups (P > 0.05) (Figure 2C).

Histopathological appearance of kidney tissues 24 hours after APAP injection showed focal area of coagulative necrosis and haemorrhage. Necrotic areas demonstrated ghosts of renal tubules, casts and lymphocytic infiltrates. The necrotic areas were sharply separated from the adjacent normal renal tissue. However, no such changes were evident in the kidneys of mice treated with 10% Pleurotus ostreatus (Figure 2D).

**Figure 3:**
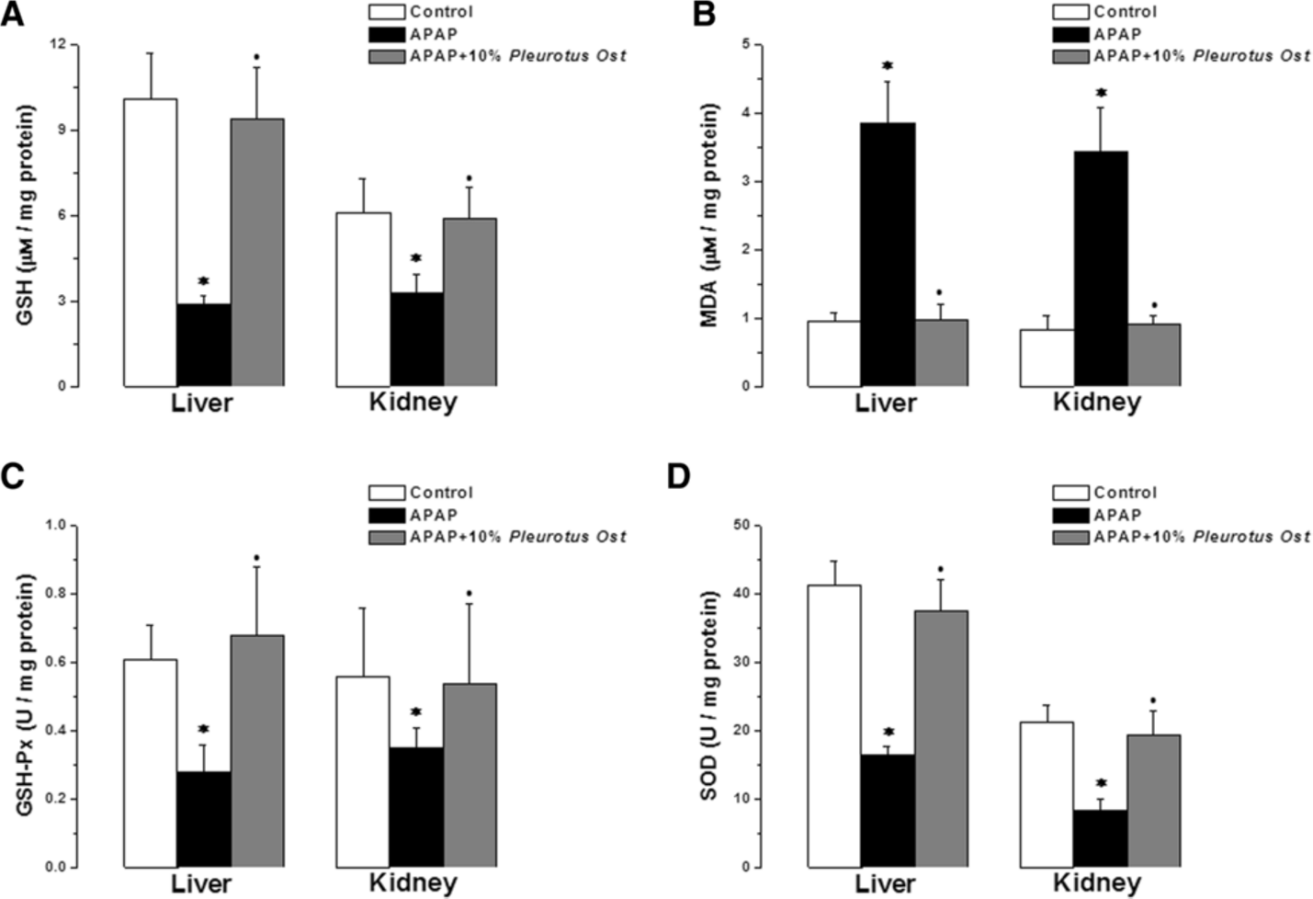
**Anti-oxidant properties of Pleurotus ostreatus. (A)** Liver and kidney GSH levels in control (white column), APAP treated (black column) and APAP + 10% Pleurotus ostreatus treated (grey column) groups. **(B)** Liver and kidney MDA levels in control (white column), APAP treated (black column) and APAP + 10% Pleurotus ostreatus treated (grey column) groups. **(C)** Liver and kidney GSH-Px levels in control (white column), APAP treated (black column) and APAP + 10% Pleurotus ostreatus treated (grey column) groups. **(D)** Liver and kidney SOD levels in control (white column), APAP treated (black column) and APAP + 10% Pleurotus ostreatus treated (grey column) groups. (Significant = p < 0.05, *significant when compared to the control group, • significant when compared to the APAP treated group. Number of mice = 10/group).

Hepatic GSH was significantly lower in the APAP group when compared to the control group (2.9 ± 0.32 vs 10.1 ± 1.6 μM/mg protein, P < 0.05). Hepatic GSH in APAP + Pleurotus ostreatus group was significantly higher than in the APAP group (9.16 ± 0.49 μM/mg protein, P < 0.05). There was no statistically significant difference in hepatic GSH between APAP + Pleurotus ostreatus and control groups (P > 0.05). Renal GSH was significantly lower in the APAP group compared to the control group (3.3 ± 0.64 vs 6.1 ± 1.2 μM/mg protein, P < 0.05). Renal GSH in APAP + Pleurotus ostreatus group was significantly higher than in the APAP group (5.9 ± 1.1 μM/mg protein, P < 0.05). There was no statistically significant difference in renal GSH between APAP + Pleurotus ostreatus and control groups (P > 0.05) (Figure 3A).

Hepatic MDA was elevated significantly in the APAP group when compared to the control group (3.86 ± 0.61 vs 0.97 ± 0.12 μM/mg protein, P < 0.05). Hepatic MDA in APAP + Pleurotus ostreatus group was significantly lower than in the APAP group (0.99 ± 0.23 μM/mg protein, P < 0.05). There was no statistically significant difference in hepatic MDA between APAP + Pleurotus ostreatus and control groups (P > 0.05). Renal MDA was significantly elevated in the APAP group when compared to the control group (3.45 ± 0.65 vs 0.83 ± 0.21 μM/mg protein, P < 0.05). Renal MDA in APAP + Pleurotus ostreatus group was significantly lower than in the APAP group (0.91 ± 0.13 μM/mg protein, P < 0.05). There was no statistically significant difference in renal MDA between APAP + Pleurotus ostreatus and control groups (P > 0.05) (Figure 3B).

Hepatic GSH-Px activity was significantly lower in the APAP group when compared to the control group (0.29 ± 0.08 vs 0.61 ± 0.1 U/mg protein, P < 0.05). Hepatic GSH-Px activity in APAP + Pleurotus ostreatus group were significantly higher than in the APAP group (0.60 ± 0.2 U/mg protein, P < 0.05). There was no statistically significant difference in hepatic GSH-Px activity between APAP + Pleurotus ostreatus and control groups (P > 0.05). Renal GSH-Px activity was significantly lower in the APAP group when compared to the control group (0.35 ± 0.06 vs 0.56 ± 0.02 U/mg protein, P < 0.05). Renal GSH-Px activity in APAP + Pleurotus ostreatus group was significantly higher than in the APAP group (0.54 ± 0.23 U/mg protein, P < 0.05). There was no statistically significant difference in renal GSH-Px activity between APAP + Pleurotus ostreatus and control groups (P > 0.05) (Figure 3C).

Hepatic SOD activity was significantly lower in the APAP group when compared to the control group (16.4 ± 1.3 vs 41.3 ± 3.5 U/mg protein, P < 0.05). Hepatic SOD activity in APAP + Pleurotus ostreatus group was significantly higher than in the APAP group (37.6 ± 4.5 U/mg protein, P < 0.05). There was no statistically significant difference in hepatic SOD activity between APAP + Pleurotus ostreatus and control groups (P > 0.05). Renal SOD activity was significantly lower in the APAP group when compared to the control group (8.4 ± 1.6 vs 21.3 ± 2.4 U/mg protein, P < 0.05). Renal SOD activity in APAP + Pleurotus ostreatus group was significantly higher than in the APAP group (19.3 ± 3.6 U/mg protein, P < 0.05). There was no statistically significant difference in renal SOD activity between APAP + Pleurotus ostreatus and control groups (P > 0.05) (Figure 3D).

**Figure 4:**
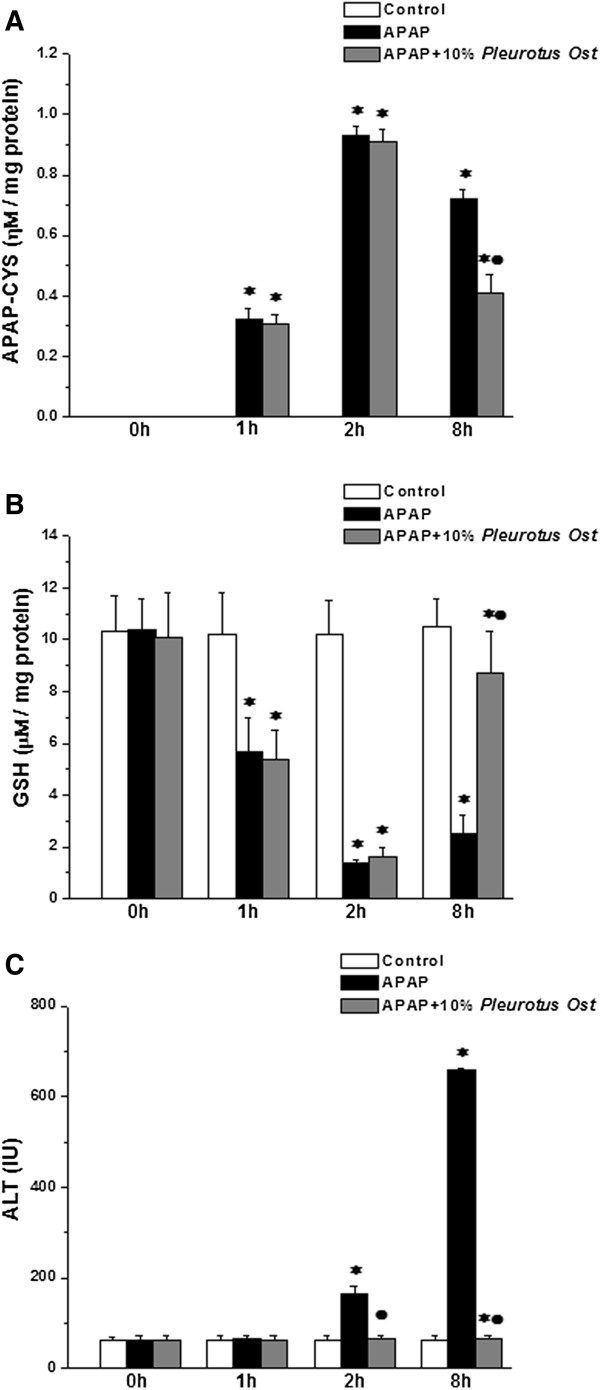
**Effects of Pleurotus ostreatus on APAP-CYS adduct formation and GSH depletion. (A)** APAP-CYS adduct level in liver tissue homogenate at 0, 1, 2 and 8 hours post APAP or saline pre-treatment in control (white column), APAP treated (black column) and APAP + 10% Pleurotus ostreatus treated (grey column) groups. **(B)** GSH level in liver tissue homogenate at 0, 1, 2 and 8 hours post APAP or saline pre-treatment in control (white column), APAP treated (black column) and APAP + 10% Pleurotus ostreatus treated (grey column) groups. **(C)** Serum ALT levels at 0, 1, 2 and 8 hours post APAP or saline pre-treatment in control (white column), APAP treated (black column) and APAP + 10% Pleurotus ostreatus treated (grey column) groups. (Significant = p < 0.05, *significant when compared to the corresponding control group at the same time point, • significant when compared to the corresponding APAP treated group at the same time point. Number of mice = 5/group).

In a separate experiment, 5 mice from each group were scarified just before (0), or after 1, 2 and 8 hours following APAP or vehicle treatment. APAP-CYS adducts and GSH depletion was measured in liver tissue homogenate, while hepatocyte injury was monitored by measuring serum ALT (Figure 4). Pre-treatment with Pleurotus ostreatus did not alter the metabolic activation of APAP as indicated by the insignificant change in APAP-CYS adducts formation at 1, 2 and 8 (Figure 4A). Simultaneously, Pleurotus ostreatus treatment restored APAP-induced GSH depletion 8 hours after APAP treatment (Figure 4B). Hepatocyte injury was only evident after 8 hours following APAP treatment as indicated by serum ALT level (Figure 4C).

## Discussion

Pleurotus ostreatus or its constituents have been reported to possess potent antioxidant, antihypercholesterolemic, immunomodulatory and anticancer properties [22–24]. However, potential hepatoprotective or nephroprotective effects and the possible involvement of antioxidant properties as the underlying mechanism have not been reported. Up to our knowledge, we report here for the first time that Pleurotus ostreatus has hepatoprotective and nephroprotective properties, as evidenced by the significant inhibition of APAP-induced changes in liver and kidney histopathology, biochemical parameters, antioxidant enzymatic activities, and lipid peroxidation products. We further show that the antioxidant properties may, at least in part, elucidate the underlying mechanism.

In the present study we demonstrated that the administration of high doses of paracetamol significantly increased serum levels of acute liver damage indicators. The serum levels of GDH, ALT and AST were significantly elevated following paracetamol administration. GDH is a key enzyme in amino acid oxidation and a potential biomarker of drug-induced hepatic toxicity [38]. In common with GDH, serum ALT is considered to be a significant indicator of acute liver damage [39]. These enzymes are present in the hepatocyte cytoplasm; therefore, damaged hepatocytes release their contents including GDH, ALT and AST into the extracellular space. The released enzymes ultimately enter the circulation and thereby increase the serum levels. Treatment with Pleurotus ostreatus protected the liver against paracetamol induced hepato-cellular injury. This was evident by the decrease in serum GDH, ALT and AST activities. The observed hepato-protective effect might be a consequence of the amelioration of the underlying mechanisms by which APAP cause cellular damage, with subsequent suppression of the leakage of these enzymes into the blood.

Our results also revealed significant renal impairment in animals treated with paracetamol, demonstrated by the increase in serum creatinine and BUN, and urinary KIM-1 levels. High doses of paracetamol have been shown to cause acute and chronic renal failure in experimental animals. The mechanism involved included deficits in the antioxidant defense mechanisms, and lipid peroxidation in renal tissue [40]. Recent work has demonstrated the potential role of KIM-1 as a sensitive and specific tissue biomarker. KIM-1 is thought to improve the early detection of acute kidney injury following the exposure to nephrotoxic compounds [41]. In the present study, we showed evidence for potential nephro-protective properties of Pleurotus ostreatus. Administration of Pleurotus ostreatus significantly reduced the increased serum creatinine and urinary KIM-1 to normal levels. Our findings, thus far, showed that Pleurotus ostreatus can oppose the injurious effects caused by high doses of APAP in the kidneys as well as the liver.

Histopathology findings confirmed the protective effect of Pleurotus ostreatus against APAP-induced liver and kidney damage. The histological appearance of the liver and kidney in the control group appeared normal. APAP treatment caused centrilobular necrosis, fatty changes (steatosis) and scattered lymphocytes infiltrate in hepatic parenchyma. Administration of APAP provoked renal proximal tubular coagulative necrosis and hemorrhage. It has been reported previously that APAP over-dose causes ultrastructural changes in the liver and kidneys [42, 43]. Following Pleurotus ostreatus administration, as shown in Figures 1D and 2D, the majority of liver and kidney tissues preserved their normal architecture with minimal inflammatory changes. Based on our findings, it is clear that of Pleurotus ostreatus can avert APAP-dependent cellular damage, thus preserving both the morphology and the function of liver and the kidneys. We were excited then to elucidate the underlying mechanisms.

Considerable progress has been made in animal models toward understanding the mechanisms of APAP toxicity. The majority of the therapeutic dose (>90%) of APAP is glucuronidated or sulfated and then excreted. A small percentage is metabolized by cytochrome P450 enzymes (CYP), in both the liver and the kidney, to the reactive intermediate N-acetyl-p-benzoquinone imine (NAPQI), which is readily detoxified by conjugation with glutathione (GSH) [44, 45]. From rodent studies, we know that higher doses of paracetamol saturate the glucuronidation and sulfation pathways, resulting in formation of excess NAPQI. The additional reactive metabolite depletes liver GSH and binds to proteins [46, 47]. Toxic doses of APAP could cause changes in the morphology and function of liver mitochondria [48, 49]. It was suggested that NAPQI binding to mitochondrial proteins leads to mitochondrial oxidative stress. It is now known that this causes the mitochondrial membrane permeability transition (MPT) pore opening, matrix swelling, and outer membrane lysis in rodent models [42, 50–52]. The permeabilization and lysis result in the release of apoptosis-inducing factor (AIF) and endonuclease G (EndoG) from mitochondria. These endonucleases translocate to nuclei and cause nuclear DNA fragmentation. Proapoptotic proteins, including cytochrome c and Smac/DIABLO, are also released. The end result is centrilobular hepatocyte necrosis and liver failure [8].

Oxidative stress has been suggested to play a critical role in cellular toxicity, as well as the pathophysiology several diseases. When the generation of ROS overcomes the antioxidant capacity, the free radicals can then interact with endogenous macromolecules and alter the cellular functions and even integrity. In the present study, high doses of APAP caused a significant rise in MDA and reduction in GSH levels in mice hepatic and renal tissues, with simultaneous inhibition of the antioxidant enzymes GSH-Px and SOD. Lipid peroxidation is a well-established mechanism of cellular injury. Lipid hydroperoxides are byproducts of lipid peroxidation, and increased levels of lipid peroxidation products are associated with a variety of chemical-induced toxicities including APAP. Lipid hydroperoxides are known to cause cellular injury by inactivation of membrane enzymes and receptors, depolymerizaton of polysaccharide, as well as protein cross linking and fragmentation [8]. A rapid depletion of GSH and lipid peroxidation has been also reported in both liver [53] and kidney [40] of animals treated with high doses of paracetamol. Paracetamol toxicity in the liver is mainly mediated by the covalent binding of NAPQI, the reactive metabolite of paracetamol, to sulfhydryl groups of GSH, and other cellular proteins and their subsequent oxidation. Overproduction of free radicals in the paracetamol treated mice may have triggered lipid peroxidation, and consequently increased MDA contents. This may also explain the diminished GSH contents; as to combat the increased formation of free radicals GSH stores might have been depleted. Decrease in GSH content can simultaneously decrease the activities of antioxidant enzymes such as GSH-Px, SOD and glutathione-S-transferase (GST) [4]. Administration of Pleurotus ostreatus significantly ameliorated the paracetamol-induced increase in MDA level and depletion of GSH contents toward normal values, with restoration of GSH-Px and SOD normal activities.

We validated the anti-oxidant properties of Pleurotus ostreatus by measuring APAP protein adducts formation and GSH levels in liver tissue homogenate. It is well known that APAP metabolic activation results in the formation of the reactive metabolite NAPQI, which reacts with GSH and with cysteine (CYS) residues on proteins [9]. Therefore, early GSH depletion kinetics (0–1 hour) may provide a logical proof on whether treatment with Pleurotus ostreatus prevents the metabolic activation of APAP or not. As illustrated by the kinetics (Figure 4), there was no significant difference in GSH depletion between mice treated with APAP and vehicle or APAP and Pleurotus ostreatus 60 min after APAP overdose suggesting that Pleurotus ostreatus had no impact on the metabolic activation of APAP. However, later kinetics (8 hours) demonstrated that Pleurotus ostreatus resulted in substantial reduction in overall protein adduct formation suggesting that an anti-oxidant incident opposed APAP-induced oxidative stress. Characterization of the mushroom extract (Table 1) revealed the presence of both essential and non-essential amino acids as tabulated (Table 1). Tyrosine, histidine, lysine and tryptophan, are generally accepted as antioxidants. Methionineis is a sulfur-containing antioxidant amino acid with well established clinically relevant anti-oxidant properties [54]. The presence of all these amino acids provides Pleurotus ostreatus with substantially a potent antioxidant capacity.

**Table 1:** **The macro-components (g/100 g dried extract) and amino acids constituents (mg/g weight) of dried** ***Pleurotus ostreatus***

Macro-components	g/100 g dried extract
Water	5.23 ± 0.29
Protein	28.15 ± 0.46
Fat	0.38 ± 0.02
Total Dietary Fiber	23.40 ± 0.05
Carbohydrates	49.30 ± 0.13
Ash	4.90 ± 0.05
**Amino acids constituents**	**w/w% protein**
Alanine	7.66
Arginine	12.66
Glutamic acid	19.01
Methionine^*^	2.52
Lecuine^*^	4.93
Isoleucine^*^	2.45
Aspartic acid	9.78
Glycine	4.93
Histidine^*^	2.88
Lysine^*^	3.50
Histidine	2.70
Norvaline	0.66
Phenylalanine^*^	3.58
Proline	2.48
Serine	5.40
Valine^*^	5.40
Threonine^*^	5.00
Tryptophan^*^	1.39
Tyrosine	3.07

^*^Essential amino acid.

## Conclusion

In summary, mitochondria are prominent targets for the toxicity of several molecules, including APAP. Mitochondrial dysfunction results in the impairment of energy metabolism and an intracellular oxidative stress with excessive formation of ROS. The antioxidant properties of Pleurotus ostreatus opposed mitochondrial dysfunction, and protected the liver and kidney tissues against APAP-induced acute inflammation.
